# Relationship between susceptibility of Blackface sheep to *Teladorsagia circumcincta* infection and an inflammatory mucosal T cell response

**DOI:** 10.1186/1297-9716-43-26

**Published:** 2012-03-28

**Authors:** Anton G Gossner, Virginia M Venturina, Darren J Shaw, Josephine M Pemberton, John Hopkins

**Affiliations:** 1 The Roslin Institute & R(D)SVS, University of Edinburgh, Easter Bush, Midlothian, EH25 9RG, UK; 2 Institute of Evolutionary Biology, School of Biological Sciences, University of Edinburgh, Edinburgh, EH9 3JT, UK

**Keywords:** Sheep, Nematode, *Teladorsagia circumcincta*, Breeding values, T cells, Cytokines

## Abstract

*Teladorsagia circumcincta* is the most economically important gastrointestinal (abomasal) nematode parasite of sheep in cool temperate regions, to which sheep show genetically-varying resistance to infection. Lambs, from parents with genetic variation for resistance*,* were trickle infected with L3 larvae over 12 weeks. 45 lambs were identified with a range of susceptibilities as assessed by: adult worm count at post mortem, faecal egg count (FEC) and IgA antibody levels. This project investigated the correlation of T cell cytokine expression and resistance to infection at the mature stage of response, when the resistant lambs had excluded all parasites.

Histopathology showed only minor changes in resistant animals with a low level lymphocyte infiltration; but in susceptible lambs, major pathological changes were associated with extensive infiltration of lymphocytes, eosinophils and neutrophils.

Absolute quantitative RT-qPCR assays on the abomasal lymph node (ALN) revealed a significant positive correlation between IL6, IL21 and IL23A transcript levels with adult worm count and FEC. IL23A was also negatively correlated with IgA antibody levels. Significantly positive correlation of TGFB1 levels with adult worm count and FEC were also seen in the abomasal mucosa. These data are consistent with the hypothesis that the inability to control L3 larval colonization, adult worm infection and egg production is due to the activation of the inflammatory Th17 T cell subset.

## Introduction

The dominant gastrointestinal parasite of farmed sheep in cool temperate regions is the abomasal strongylid nematode *Teladorsagia circumcincta*[1,2]. With the progression of resistance to broad spectrum anthelmintics [3] and the impracticality of many pasture management systems [4] there is a need to investigate alternative methods of parasite control. The greatest susceptibility to *T. circumcincta* infection occurs with weaned lambs during their first grazing season [5]. However, many sheep eventually control worm development and egg production through the acquisition of protective immunity [6,7], largely through the generation of parasite specific IgE and IgA antibodies [8-10], which act to reduce worm length and fecundity and exclude larval colonization.

Our data and others [11,12] show a significant linkage between IgA antibody levels, adult worm numbers, worm length and faecal egg counts (FEC). Furthermore, individual measures of FEC are repeatable over time and heritable by six months [13] indicating the feasibility of selective breeding for reduced FEC. The original breeding programmes for the development of resistant and susceptible lines to *Haemonchus contortusTrichostrongylus colubriformis* as well as *T. circumcincta* were based on FEC [14-16]. More recently, microsatellite and quantitative trait locus (QTL) analyses [17,18] have identified a number of markers associated with resistance, including alleles of Ovar-DRB1 [19] and IFNG [20].

CD4^+^ T cell depletion abrogated nematode resistance of selected sheep lines [21], identifying this T cell subset as critical in the immunological control of nematode development and egg production. Murine models using *Heligmosomoides polygyrus* and *Nippostrongylus brasiliensis* have linked high levels of interleukin 4 (IL4), IL10 and IL13 with nematode resistance and high IL2 and interferon γ (IFNγ) with susceptibility [22,23]. Similar studies in sheep using acute challenge with *T. circumcincta* or *H. contortus*, of infected/reinfected (‘immune’) animals or selected resistant and susceptible lines has confirmed the murine data identifying the type 2 (Th2) polarized immune response [24-26] in the development of protection/resistance probably through the promotion of mucosal mast cell development and the production of IgE and IgA antibodies. However, it is unclear whether the generation of a type 1 (Th1) response is associated with susceptibility and that resistance/susceptibility to gastrointestinal nematodes is simply a matter of Th1/Th2 dichotomy [27]. The CD4^+^/CD25^+^/Foxp3^+^ regulatory T cell subset (Treg) has been shown to be critical for the clinical outcome of helminth infection. Resistance to helminths in mice seems to be determined by a balanced Th1/Th2/Treg response; unbalanced modified Th2 (high Th2/Treg) and uncontrolled Th1 (high Th1) results in persistent infection and clinical disease [22,28]. More recently it has been found that the Th17 CD4^+^ T cell subset also plays an important role in human and mouse inflammatory bowel diseases as well as host responses to parasites [29,30]. Indeed, there seems to be a reciprocal development of Treg and Th17 cells in autoimmune or bacteria-associated inflammatory diseases [31-33].

It is clear that the immunology of nematode infection is specific for both helminth and host species and strains. In mice, a highly polarized Th2 response controls *H. polygyrus* whereas *Schistosoma mansoni* induces more mixed Th1 and Th2 responses [23]. Furthermore, *Trichuris muris* is expelled from Balb/c mice by a polarized Th2 response but the AKR strain become chronically infected in the presence of a Th1 response [22]. In contrast, the immunology of *T. circumcincta* control in sheep seems to be distinct from these murine models [25] in that IFNγ expression was unaffected by infection of either ‘immune’ or ‘naïve’ lambs.

To examine the immunology of parasite resistance in sheep we exploited naïve Blackface lambs with diversity in their predicted genetic resistance to *T. circumcincta*, which were trickle-infected with L3 larvae to simulate natural infection [11]. This schedule resulted in animals with a range of susceptibilities that reflected the nature and magnitude of the mature immune response, three months after the first infection. Adult worms were highly aggregated in few lambs and absent in resistant lambs, while the early arrested larvae (EAL4) were uniformly distributed across the flock. We tested the hypothesis that resistance/susceptibility to *T. circumcincta* is associated with differential activation of Treg and Th17 T cells as well as the interaction between Th1 and Th2 subsets. These four subsets were investigated by measurement of the transcripts of their characteristic markers and effector cytokines and quantitative expression correlated with individual traits of resistance and susceptibility.

## Materials and methods

### Animals and experimental design

Animals were 55 female Blackface lambs, housed in worm-free conditions; the parents of which belonged to a Blackface flock previously used for quantitative genetic and QTL analyses of FEC (as eggs per gram wet weight faeces) [14]. Ten lambs were chosen as controls and were sham infected, and 45 lambs were experimentally infected with ~2,300 infective L3 *T. circumcincta* larvae three times a week for 12 weeks and sacrificed after 13 weeks; lambs were 10–13 weeks old at the start of infection. The uninfected controls were twins of lambs (rank number 5, 7, 22, 31, and 41) in the infected group. At *post mortem* ten infected lambs had no detectable adult worms in the total abomasal contents, while the other infected lambs had a range of adult worm counts up to 11,300 (Additional file 1). Other parameters of parasite infection and immunity were correlated with these differences in adult worm numbers: resistant lambs had low/no FEC, high IgA antibody levels and high body weight; susceptible lambs had high FEC, low IgA antibody levels and low body weight. Details of the animals, infection protocols, trait and population genetic analyses have been described previously [11].

Abomasal lymph nodes (ALN) and abomasal mucosa were removed immediately post mortem and 5 mm blocks stored in RNAlater (Ambion, Huntingdon, UK) at −80°C. Tissues for histology were fixed in zinc salts fixative [34]; 5 μm sections from the paraffin wax-embedded tissue were stained with haematoxylin and eosin. Animal experiments were approved by University of Edinburgh Ethical Review Committee and conducted under an Animals (Scientific Procedures) Act 1986 Project Licence.

### RNA extraction and cDNA synthesis

Total RNA was isolated using the Ribopure Kit (Ambion) for abomasal lymph node and RNeasy (Qiagen, Crawley, UK) for abomasal mucosa samples according to the manufacturers’ instructions; all samples were DNase I digested using Turbo DNA-free (Ambion). The quality and integrity of the RNA samples were analyzed using the Agilent® 2100 bioanalyzer (Agilent Technologies); all had an RNA Integrity Number of >6.

### Cloning of ovine gene fragments

The sequences of sheep IL7R, IL17A, IL21, IL23A and IL25 were not available at the start of the study; therefore partial sequences were selected using Primer3 Plus [35] and Net Primer (http://www.premierbiosoft.com/netprimer/index.html) based on the bovine sequences (Additional File 2A). All selected primer sequences were then checked for possible cross-hybridization using NCBI-BLAST (http://blast.ncbi.nlm.nih.gov/Blast.cgi).

cDNA was synthesized from 0.5 μg total RNA with Superscript III reverse transcriptase (Invitrogen, Paisley, UK) using an oligo-dT primer. RT-PCR was performed using primers to amplify partial cDNA sequences of sheep genes (Additional file 2A). The reaction consisted of 0.5–2.0 μl of cDNA, 5 μl (10x) PCR buffer (Promega); 1 μl dNTP mix (Promega); 1 μl of each primer at 0.5 μM, MgCl_2_ varied from 2–4 mM and nuclease free water to a final volume of 50 μl. The mixture was incubated at 95°C for 2 min prior to the addition of 0.4 μl Taq DNA polymerase (5U/μl:Roche). Reactions were: 30–35 cycles of 60 s at 94°C, annealing for 60 s at varying temperatures (Additional file 2A); and 2 min at 72°C followed by a final extension at 72°C for 5–7 min. PCR products were analysed by agarose gel electrophoresis, visualized by gel red/UV transillumination, purified using MinElute PCR Purification Kit (Qiagen) and cloned into pGEM-T Easy (Promega, Southampton, UK). A random selection of clones were sequenced on both strands using T7 and SP6 sequencing primers in separate reactions with BigDye® Terminator v3.1 Cycle Sequencing Kit (Applied Biosystems, Warrington, UK). Three independent sequences were obtained for each clone and primers (Additional file 2B) for quantitative real-time RT-PCR (RT-qPCR) were selected from these sequences, using Primer3 Plus. RT-qPCR for all other transcripts was performed using primers designed from published sequences or as described previously [36,37].

### Quantitative real-time PCR analysis

qPCR was carried out in a 10 μl final volume containing 5 μl template cDNA (diluted 1/10 to 1/40) or linearized plasmid DNA, 1.0 μl 10x buffer, 0.35 μl SYBR Green I (BioGene, Cambridge, UK), 0.2 μl dNTP mix (10 μM each dNTP), optimized Mg^2+^ (2.0–3.5 mM) and primer concentrations (0.25–1.0 μM), 0.375 U FastStart Taq DNA polymerase (Roche Applied Science) added to nuclease-free water. All reactions were prepared using a CAS-1200™ Precision Liquid Handling System and performed on the Rotor-Gene™ 3000 or Rotor-Gene Q (Qiagen). The amplification profile used was the same for each gene except for the annealing temperature; 5 min at 94°C, followed by 40 cycles of 20 s at 94°C, 20 s at optimized annealing temperature for particular primer set (Additional file 2B) and 20 s at 72°C, followed by dissociation curve analysis to confirm a single gene product. Each sample was assayed in triplicate within a run and a no-template control was included in all runs.

Both relative and absolute copy number expression levels were quantified in three separate RT-qPCR runs, each time using cDNA from a different RT reaction. To derive the copy number of the target sequence in unknown samples a standard curve (linearized plasmid DNA) was used with a dynamic range that spanned at least five orders of magnitude. Copy numbers were calculated by; the molecular weight of vector plus insert (M) = size of plasmid and insert size bp × 660 g/mol per bp. Number of molecules per ng = ((1 × 10 × ^-9^)/(M g/mol)) × (6.023 × 10^23^molecules/mol). Linearity and efficiency of qPCR amplification was determined for each primer pair using a standard curve generated by a dilution series of a pool of sample cDNAs for each tissue. All reactions had a PCR efficiency of > 90% and correlation coefficients were R^2^ >0.98 (Additional file 2B).

Gene expression levels were calculated in GenEx version 5.3.4.157 (http://www.multid.se.) using the comparative 2-(ΔΔ Cq) method and normalized to the geometric mean of the stably-expressed reference genes (*SDHA* and *YWHAZ*) as selected using GeNorm v3.4 [38] and the NormFinder Microsoft Excel applet. Fold changes were calculated from delta Cq values using GenEx. Normalized copy numbers were obtained using the normalization factor determined by GeNorm. The expression levels were normalized by dividing the copy number derived from the standard curve by the calculated normalization factor for each individual sample.

### Statistical analysis

Linear mixed effect models were run in which the identity of individual sheep was entered as a random effect, RT reactions and RT-qPCR replicates were fixed effects. Statistical differences between groups were determined by one-way ANOVA with Tukey-Kramer’s post-hoc test for multiple pairwise comparison analysis, in GenEx. The correlations between transcript levels and both faecal egg counts and adult worm burdens were analyzed with Spearman’s correlation coefficient (ρ), due to the over-dispersed parasite number distributions, using R (v 2.10.1 © 2009 R Foundation for Statistical Computing). P-values less than 0.05 were considered statistically significant.

## Results

### Histopathology of abomasal mucosa

**Figure 1:**
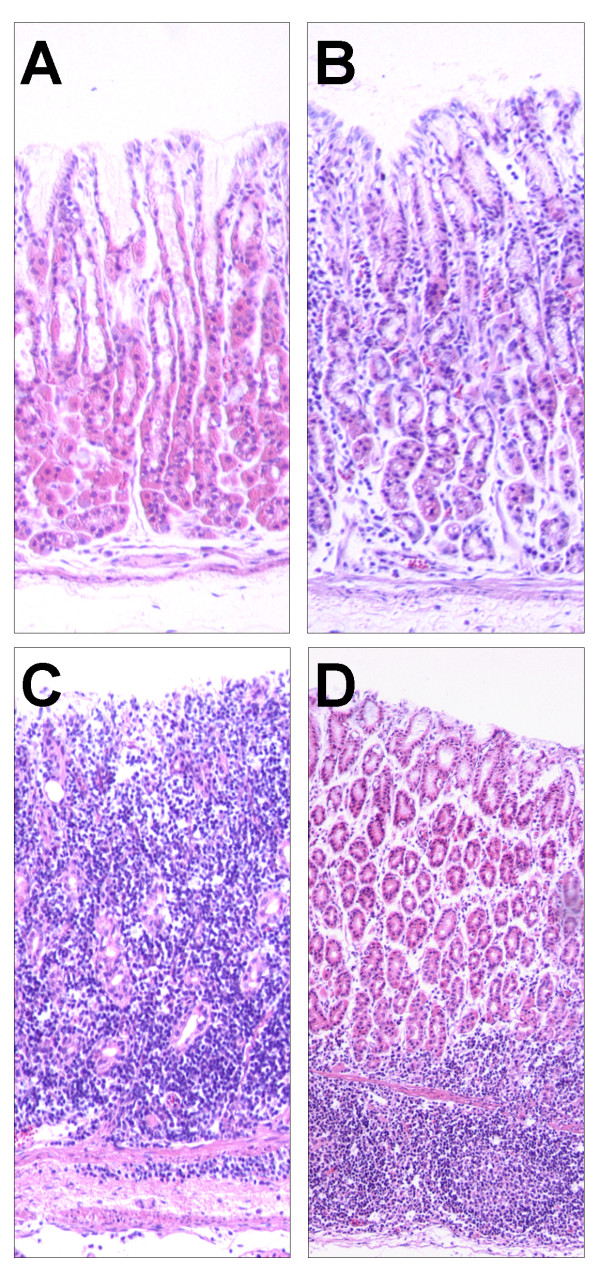
Histopathology of abomasum H&E stained sections (x100) of abomasum from uninfected control (A), resistant (B) and susceptible lambs (C and D).

Histopathology of the abomasum was performed on fifteen lambs (Additional file 1) comprising five uninfected controls, five with the lowest adult worm count and FEC (resistant; mean adult worm count = 0, FEC = 0) and five with the highest adult worm count and FEC (susceptible, mean adult worm count = 6800, FEC = 500). In comparison to the uninfected control group (Figure 1A), only minor pathological changes were noted in resistant animals with a low level lymphocytic infiltrate and few eosinophils and neutrophils associated with the gastric glands (Figure 1B); nematodes were not detected within the gastric glands. In the abomasal mucosa of susceptible lambs, major pathological changes were associated with extensive infiltration of lymphocytes, eosinophils and neutrophils in the mucosal and sub-mucosal layers (Figure 1C). Oedema was also observed in many areas with vacuolation in the mucosal layer surrounded with lymphocytes, neutrophils, eosinophils, and cellular debris suggestive of larval migration. Lymphoid aggregates were also frequently detected in some regions of the abomasal sub-mucosa in susceptible lambs (Figure 1D).

### Comparative transcript expression between resistant and susceptible groups

Relative analysis of all fourteen genes was performed on the ALN and abomasal mucosa of the same fifteen lambs selected for histopathology (Table 1) and was performed to help select targets for future absolute quantification (transcript copy number) analysis. The transcript expression levels of IL4, IL17A and IL25 in the ALN were below the threshold levels of the assay and therefore could not be quantified accurately. Linear mixed effect analysis showed that neither the random nor fixed factors significantly influenced the between-group comparisons (data not shown). No significant differences were found with any comparison for IL2, IL7R, IL10, IL12B, IL23A and TGFB1. In contrast, significant (all P ≤0.03) differences between groups were observed for IL6, IL21 and EBI3. IL6 and IL21 expression was highest in susceptible lambs, resulting in significant fold changes (P < 0.01 for IL6; P = 0.03 for IL21) in both the S vs C and S vs R comparisons. EBI3 expression was lowest in susceptible lambs resulting in a significant −2.56 fold change in the S vs C comparison (P = 0.02) but not in the R vs C comparison; the S vs R comparison showed a −1.85 fold change and was marginally insignificant (P = 0.1). FOXP3 expression was raised in both infected groups, resulting in significant 1.68 (P = 0.04) and 1.91 (P < 0.01) fold changes in the S vs C and R vs C comparisons respectively, but not in the S vs R comparison (−1.1 fold, P = 0.59). IFNG expression was lowest in the susceptible lambs resulting in a significant −1.66 fold change (P = 0.04) in the S vs C comparison; there were no significant changes in the R vs C or S vs R comparisons.

**Table 1:** Relative quantification of mRNA transcripts in abomasal lymph node

	**Susceptible vs Control**		**Resistant vs Control**		**Susceptible vs Resistant**	
**Gene**	fold change* ± sd	**P-value**	**fold change** **± sd**	**P-value**	**fold change** **± sd**	**P-value**
IL2	1.58 ± 0.51	0.20	1.31 ± 0.66	0.60	1.32 ± 0.43	0.68
IL4	**ND**		ND		**ND**	
IL6	**2.30 ± 0.43**	**<0.01**	1.25 ± 0.45	0.88	**1.95 ± 0.36**	**<0.01**
IL7R	1.04 ± 0.28	0.10	−1.01 ± 0.54	0.96	1.17 ± 0.31	0.98
IL10	1.73 ± 0.60	0.16	1.51 ± 0.29	0.42	1.16 ± 0.40	0.78
IL17A	ND		ND		ND	
IL12B	−1.23 ± 0.25	0.74	1.15 ± 0.69	0.90	−1.25 ± 0.24	0.48
IL21	**2.06 ± 0.78**	**0.03**	−1.01 ± 0.48	0.99	**2.24 ± 0.85**	**0.03**
IL23A	1.40 ± 0.19	0.23	1.19 ± 0.36	0.76	1.22 ± 0.16	0.58
IL25	ND		ND		ND	
EBI3	**−2.56 ± 0.19**	**0.02**	−1.19 ± 0.46	0.63	−1.85 ± 0.27	0.10
FOXP3	**1.68 ± 0.33**	**0.04**	**1.91 ± 0.43**	**<0.01**	−1.11 ± 0.18	0.59
IFNG	**−1.66 ± 0.23**	**0.04**	−1.22 ± 0.22	0.40	−1.32 ± 0.29	0.38
TGFB1	1.51 ± 0.28	0.22	1.46 ± 0.49	0.16	1.08 ± 0.20	0.98

* Fold change is the ratio of normalized mean expression between groups. Means of the uninfected controls were used as the calibrator and compared with the infected animals; n = 5 for each group.

ND; not determined, IL4, IL17A and IL25 levels were outside the dynamic range of linearity of the assay and were too low to be accurately quantified and therefore valid comparisons could not be made. Bold is P ≤ 0.05.

The expression of only six genes could be determined in abomasal mucosa (Table 2), all others were below the threshold of the detection. Of those, IL7R, IL10, EBI3 and IFNG showed no significant difference between the three groups. IL6 and TGFB1 expression were highest in susceptible lambs; IL6 showed a significant (P < 0.01) 4.44 fold change in the S vs C comparison and TGFB1 showed a 2.53 fold change (P < 0.01) and a 1.88 fold change (P < 0.01) in the S vs C and S vs R comparisons respectively.

**Table 2:** Relative quantification of mRNA transcripts in abomasal mucosa

	**Susceptible vs Control**		**Resistant vs Control**		**Susceptible vs Resistant**	
Gene	fold change ± sd	P- value	fold change ± sd	P-value	fold change ± sd	P-value
IL6	4.44 ± 1.91	**<0.01**	2.48 ± 0.81	0.23	1.79 ± 2.36	0.07
IL7R	−1.33 ± 0.19	0.33	1.00 ± 0.41	0.74	−1.33 ± 0.45	0.74
IL10	1.00 ± 0.30	0.66	−1.54 ± 0.18	0.19	1.53 ± 1.63	0.60
EBI3	−1.43 ± 0.40	0.33	−1.16 ± 0.35	0.70	−1.22 ± 1.12	0.78
IFNG	2.03 ± 1.30	0.89	1.44 ± 0.76	0.94	1.41 ± 0.70	0.70
TGFB1	2.53 ± 0.35	**<0.01**	1.35 ± 0.32	0.41	1.88 ± 0.12	**<0.01**

Bold is P ≤ 0.05. n = 5 for each group

### Association between phenotypic variation and transcript copy number

IL6, IL21, IL23A, EBI3 and TGFB1 were chosen for copy number measurement in the ALN of all 55 animals. These were analysed in four groups: ten uninfected controls; a resistant group comprising fifteen with the lowest adult worm count and FEC (mean adult worm count = 59, mean FEC = 1.67); the fifteen most susceptible sheep with the highest adult worm count and FEC (mean adult worm count = 5167, mean FEC = 288); and fifteen intermediate sheep with mean adult worm count = 1508, mean FEC = 82 (Additional file 1).

**Figure 2:**
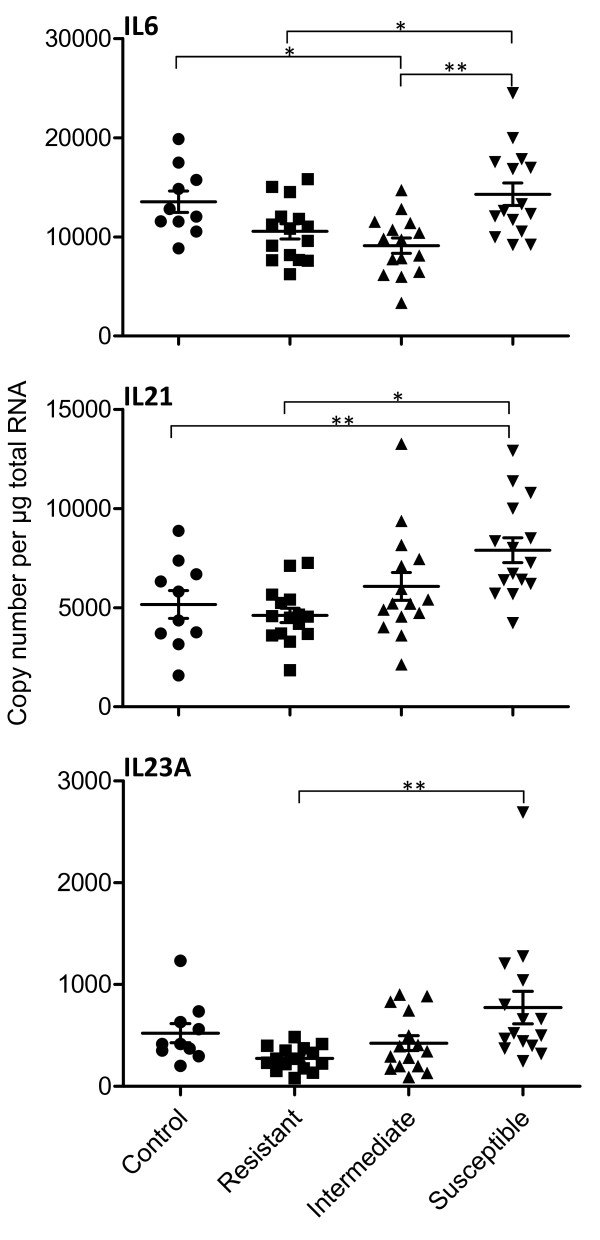
Cytokine transcript expression in the abomasal lymph node of*T. circumcincta*infected sheep copy number per μg total RNA. * P ≤ 0.03 and ** P ≤ 0.01 for the individual comparisons. Control, n = 10; Resistant (FEC < 10), n = 15; Intermediate (FEC 10 – 219), n = 15; Susceptible (FEC > 220) n = 15. Error bars are means ± SD.

Only IL6, IL21 and IL23A showed significant differences in transcript expression in the ALN between any of the four groups (Figure 2). The copy number of IL6 transcripts in resistant sheep was 10550 ± 2941 per μg of total RNA, significantly lower than the susceptible lambs 14299 ± 4447 (P = 0.02). IL6 levels in intermediate lambs were 9108 ± 2983, significantly lower than both the uninfected controls 13525 ± 3398 (P = 0.02) and the susceptible group (P = 0.0001), but not significantly different to the resistant (P = 0.67) group. Both IL21 and IL23A showed a graded increase from the resistant to the susceptible groups. IL21 levels in the uninfected group were 5159 ± 2218 and in the resistant, intermediate and susceptible groups were 4611 ±1407, 6065 ± 2715 and 7894 ± 2433 respectively; IL21 transcript levels in the susceptible group were significantly higher than in the control (P = 0.01) and resistant (P = 0.02) groups. IL23A levels in controls were 522 ± 297, and in the resistant, intermediate and susceptible groups were 274 ± 115,425 ± 283 and 773 ± 618 respectively; only the resistant and susceptible comparison was significantly different (P = 0.01). The levels of EBI3 varied from 9826 ± 4404 in the susceptible group to 10781 ± 4405 in the intermediate group. The levels of TGFB1 varied from 255521 ± 34753 in the controls to 287122 ± 59198 in the resistant group. There were no significant differences between the four groups for either EBI3 (P ≥ 0.77) or TGFB1 (P ≥ 0.68).

**Figure 3:**
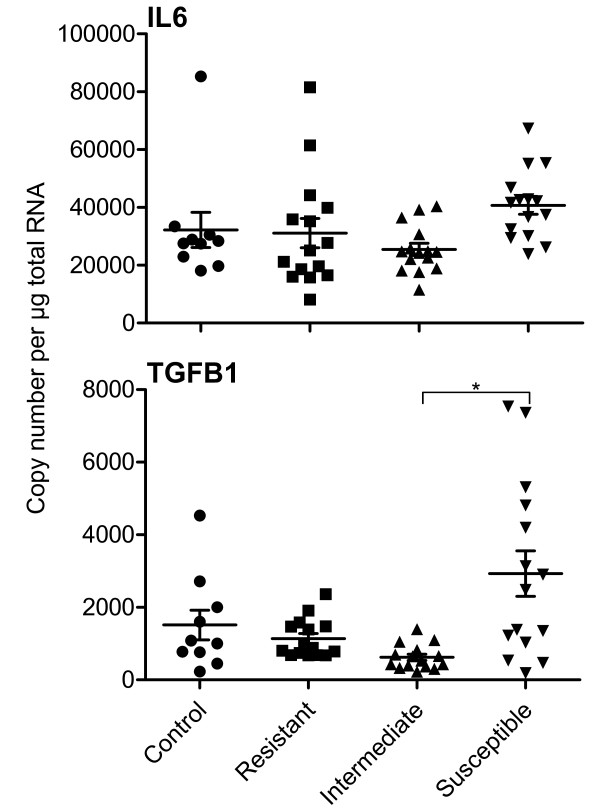
**Cytokine transcript expression in the abomasal mucosa of*T. circumcincta*infected sheep.**copy number per μg total RNA. * P ≤ 0.03 and ** P ≤ 0.01 for the individual comparisons. Control, n = 10; Resistant (FEC < 10), n = 15; Intermediate (FEC 10 – 219), n = 15; Susceptible (FEC > 220) n = 15. Error bars are means ± SD

IL6 and TGFB1 (Table 2) were chosen for copy number measurement in the abomasal mucosa of all 55 animals. The expression level of TGFB1 transcripts in the susceptible group (Additional file 3) was 40687 ± 11964 and was significantly different (P < 0.04) from TGFB1 levels in the intermediate group (25472 ± 7910). The other TGFB1 comparisons and all comparisons for IL6 showed no significant differences (Figure 3).

Spearman’s rank correlation analysis (Table 3) showed that both IL6, IL21 and IL23A transcript levels were significantly positively correlated with both adult worm count (ρ = 0.348 for IL6, ρ = 0.537 for IL21) and FEC (ρ = 0.408 for IL6, ρ = 0.651 for IL21,) but were not significantly correlated with either IgA antibody levels or body weight. IL23A was also significantly positively correlated with adult worm count (ρ = 0.378) and FEC (ρ = 0.306) and significantly negatively correlated with IgA antibody levels (ρ = −0.308) but not with body weight. EBI3 and TGFB1 were not significantly correlated with any of the four phenotypic parameters. However, TGFB1 levels in the abomasal mucosa were significantly positively correlated with both adult worm count (ρ = 0.4255) and FEC (ρ = 0.317).

**Table 3:** Correlation analysis of phenotypic parameters with cytokine transcript copy number in ALN and abomasal mucosa

	**adult worm count**		**FEC**		**IgA**		**body weight**	
	**ρ**^**a**^	**P value**	**ρ**	**P value**	**ρ**	**P value**	**ρ**	**P value**
**Abomasal lymph node**								
IL6	**0.348**	**0.019**	**0.408**	**0.0054**	−0.21	0.167	0.0665	0.664
IL21	**0.537**	**0.0001**	**0.651**	**<0.0001**	−0.135	0.377	0.031	0.838
IL23A	**0.378**	**0.0104**	**0.306**	**0.041**	**−0.308**	**0.0396**	−0.054	0.05
EBI3	0.003	0.982	−0.179	0.239	−0.164	0.283	−0.025	0.870
TGFB1	−0.128	0.402	−0.020	0.895	−0.125	0.414	0.138	0.365
**Abomasal mucosa**								
IL6	0.1417	0.3526	0.1563	0.3053	−0.2737	0.0688	−0.1979	0.1926
TGFB1	**0.4255**	**0.0036**	**0.317**	**0.034**	−0.2558	0.0899	0.0066	0.9657

Bold; significant correlation (P ≤ 0.05).

^a^ρ, Spearman’s rank correlation coefficient; n = 45 infected lambs.

## Discussion

The aim of this study was to investigate the relationship between the host immune response and parasite infection in sheep selected to have a range of predicted genetic resistance to the abomasal nematode parasite *T. circumcincta*. Naïve lambs were trickle-infected every two days over a period of 12 weeks to mimic natural infection, and analysed at a stage when the mature immune response of the resistant animals was controlling and/or eliminating recurrent parasite infection. At the same time point, the response of other animals in the group that retained mature adult nematodes and excreted numbers of parasite eggs, did not control infection [11].

The histopathology of these different animals reflected the differential response. The abomasal mucosa of resistant lambs showed only minor pathological changes, with the gastric glands empty of larvae despite the animals being repeatedly infected. These animals had high FEC until approximately day 50 post-infection, stimulating IgA antibody production [11] and parasite elimination assessed by FEC [8]. Mast cell and eosinophils seem to play a major role in the acute response to *T. circumcincta* infection [39]. However, the high levels of IgA antibody present as a result of persistent trickle-infection, and the consequent paucity of colonizing worms is a likely explanation for the low level of pathology (including few mast cells and eosinophils) in the resistant animals described in this study.

At post-mortem there were no significant changes to the expression of any cytokine transcript in either the ALN or abomasal mucosa of resistant sheep implying that this IgA antibody probably inhibited colonization of recently delivered infective L3 larvae obviating the activation of an immune or inflammatory response [40,41]. In contrast were the gross immunoinflammatory lesions in susceptible lambs, which had low/insignificant IgA antibody levels and were unable to control parasite colonization and egg production.

Previous work on T cell immunology of *T. circumcincta* focussed on acute responses in naïve and previously infected/treated yearling sheep and identified increased expression of Th2 cytokines early after infection as critical for worm expulsion [25]. Work on the related abomasal nematodes *H. contortus* in sheep [24,42] and *Ostertagia ostertagi* in cattle [10,43] as well as murine models [27,44], also linked Th2 cytokines with responses in previously-infected animals; associating the up-regulation of IL4 and IL13 with parameters of immunity including; IgE and IgA antibodies, eosinophilia and mucosal mast cells. Th2 immunity has also been implicated in the acute immune response to primary infection with *H. contortus* in the intrinsically-resistant BBB breed of sheep but not in the intrinsically-susceptible INRA breed [45]. Similarly, an acute Th2-cytokine profile is associated with control of infection in sheep lines selected for resistance to *T. colubriformis*[46].

Some of these studies also report an up-regulation or unchanged expression of the type 1 cytokines IL12 and IFNγ in previously immunized animals, implying that immunological control of these parasites is not simply a consequence of a stereotypic Th2 response [10,25]. Our data on the mature immune response supports this hypothesis. Expression levels of transcripts of the archetypical type 1 cytokines IL12 and IFNγ and the type 2 cytokines IL4 and IL10, did not correlate with resistance and susceptibility respectively. We could detect very little IL4 in any animal; while IL10 and IL12B showed no significant changes and IFNG was significantly reduced in susceptible animals.

Copy number measurements show that there was significant increase in expression of IL6, IL21 and IL23A in the susceptible group of lambs in comparison to the resistant animals and there was a significant positive correlation of IL6, IL21 and IL23A with adult worm numbers and FEC. Furthermore, there was a significant negative correlation of IL23A levels with IgA antibody levels. Comparative analysis of the induced Treg transcriptional regulator FOXP3 [47] showed that both the susceptible as well as the resistant animals have significantly greater expression than the uninfected controls. These data are consistent with the hypothesis that susceptibility is related to the development and activation of the inflammatory Th17 subset of CD4^+^ T cells [48,49] and a possible imbalance between the Th17 and Treg subsets [32,50], possibly inhibiting the generation of protective IgA antibody. Supporting this hypothesis are our findings from digital gene expression experiments [51] that showed significantly higher expression of HLX and TGFBR1 transcripts in the susceptible group of lambs; both are associated with differential T cell development [52].

Th17 and Treg cells are distinct from the Th1 and Th2 cell lineages [48] and are particularly associated with mucosal, especially gastrointestinal, surfaces [31] where they function to protect the epithelium from invading pathogens. The development of both subsets is dependent of TGFβ; what determines the balance between Th17 and Treg seems to be IL6 [53] which inhibits Treg and promotes Th17. The induction of Th17 from naïve T cells also seems to be controlled by IL23A released by innate immune cells, including macrophages and dendritic cells [54,55]. Th17 cells have a major inflammatory function through the expression of IL17A and IL21 as well as IL1β, IL6 and TNFα [32,56]. IL21 produced by Th17 cells seems to act in a positive feedback loop to promote further Th17 differentiation [32]. The susceptible lambs had the highest levels of IL6, IL21 and IL23A transcripts in the ALN while retaining consistent levels of TGFB1 (TGFβ). Furthermore, significantly higher levels of TGFB1 were found in the abomasal mucosa of susceptible lambs than in the other groups. An argument against this hypothesis is that IL17A transcripts could not be detected in any sheep in this experiment. However the sheep tissues were from persistently infected animals undergoing mature immune responses and this finding is not inconsistent with data from mice where IL17A is only produced in acute responses early after activation, and declines within the first week [57]. Treg cells develop in the absence of IL6 and function to control the inflammatory reaction by the expression of the regulatory cytokine IL10 [28]. In relation to the balance between Th1 and Th17, high levels of the Th1 cytokine IFNγ suppress Th17 production [58]. In our experiments, the susceptible lambs had a significantly reduced level of IFNG transcripts, possibly explaining the high Th17 in these animals.

In conclusion, we have examined the immunological basis of resistance and susceptibility of lambs to persistent infection with the common abomasal parasitic nematode, *T. circumcincta*. Histopathology showed only mild pathological changes to the abomasal mucosa of resistant lambs but gross lymphoid infiltration and inflammation in the mucosa and sub-mucosa of infected susceptible animals. Associated with these inflammatory changes were significantly higher levels of IL6, IL21 and IL23A transcripts in the abomasal lymph nodes, and TGFB1 in the mucosa. These data are consistent with the hypothesis that susceptibility, and therefore inability to control parasite colonization and egg production, is associated with increased levels of activation of the inflammatory Th17 T cell subset.

## Competing interests

The authors declare that they have no competing interests.

## Authors’ contributions

JMP and JH conceived the study, collected the samples and were the grant holders. AGG and JH designed the experiments. AGG and VMV performed the experiments. VMV and DJS were responsible for the statistical analysis. JH drafted the manuscript. All the authors have read and approved the final manuscript.

## Supplementary Material

Additional file 1 Normalized copy numbers of cytokine transcripts in abomasal lymph nodes. Click here for file

Additional file 2 PCR primers and PCR conditions A. Primers sets based on Bos taurus sequence for amplification of sheep cytokine transcripts. B PCR primers and PCR conditions. Click here for file

Additional file 3 Normalized copy numbers of cytokine transcripts in abomasal mucosa. Click here for file
