# Supplementary material for: Relationship between susceptibility of Blackface sheep to Teladorsagia circumcincta infection and an inflammatory mucosal T cell response
Source: Vet Res. 2012 Mar 28;43(1):26. doi: 10.1186/1297-9716-43-26 (PMC3422184; doi:10.1186/1297-9716-43-26)
Supplement: Additional file 1 — Normalized copy numbers of cytokine transcripts in abomasal lymph nodes. [file 1297-9716-43-26-S1.pdf]

Additional file 1
Normalized copy numbers of cytokine transcripts in abomasal lymph nodes

Infection rank[a]	Adult worm count	FEC (eggs/g)	IgA[c]	Body Weight (kg)	Copy numbers per µg total RNA				
					IL6	IL21	IL23A	EBI3	TGFB1
Uninfected Control	mean adult worm count = 0, mean FEC = 0.								
0	0	0	0.027	35.5	14848	5812	1233	6217	277905
0	0	0	0.02	36	15740	1579	632	15224	273238
0[b]	0	0	0.177	36	11569	6316	372	10112	261563
0[b]	0	0	0.025	34.5	11545	3695	737	11085	255047
0	0	0	0.015	29	12031	3152	416	6638	264836
0[b]	0	0	0.288	27.5	19852	8863	560	11398	235090
0[b]	0	0	0.13	30	17499	4358	297	9455	219535
0[b]	0	0	0.031	36	12800	7373	202	7415	311237
0	0	0	0.146	33	8827	3762	416	8275	271048
0	0	0	0.077	32	10543	6682	350	7005	185708
Group 1 – Resistant	mean adult worm count = 59, mean FEC = 1.67.								
1[b]	0	0	1.195	30	14505	5230	398	9278	285751
2	0	0	0.63	29	6236	3697	218	5605	319857
3[b]	0	0	0.798	36	15049	3593	271	10475	382993
4[b]	0	0	0.633	28	15805	7102	352	4134	380748
5[b]	0	0	0.077	30	9593	1847	415	12614	340326
6[b]	0	0	0.373	32	7643	4567	483	9750	285727
7	0	0	0.384	25	8151	3277	374	19023	242788
8	0	0	1.695	15	9121	3667	177	11453	210377
9	0	0	1.066	27	11025	7259	329	13117	210896
10	80	0	0.856	30	7651	4549	272	6985	317280
11	100	0	0.126	29	7577	4494	224	7408	200657
12	100	0	0.547	32.5	11819	4186	134	7892	241734
13	100	25	0.154	34	12040	5403	81	7904	306587
14	200	0	0.782	27	10802	5658	232	9783	253920
15	300	0	0.706	29	11237	4642	151	5231	327194
Group 2 – Intermediate	mean adult worm count = 1508, mean FEC = 82.								
16	400	0	0.706	39	6150	4006	293	7901	247866
17	420	75	0.232	26	11377	9367	92	7268	267654
18	200	25	0.232	27.5	6463	7099	900	11941	172305
19	600	0	0.596	20	8101	5210	887	12495	185710
20	900	0	0.804	37	7826	4899	830	4309	172130
21	800	0	0.703	26	10711	5189	499	9157	198136
22	1200	0	0.142	35	10405	4737	405	16643	240921
23	1700	175	0.21	26	12801	13262	393	6642	235585

24	1200	0	0.161	28	14711	2135	746	10501	635008
25	2400	0	0.732	26	3336	5405	344	11582	190601
26	2300	175	1.51	34	11517	8153	174	3415	260633
27	2400	475	0.539	35	5971	7441	198	14135	223056
28	2400	75	0.183	31	9803	5942	131	11252	303635
29	2600	100	0.259	36	7731	3591	199	16544	315039
30	3100	125	0.468	37.5	9719	4543	282	17926	230453
Group 3 - Susceptible	mean adult worm count = 5167, mean FEC = 288.								
31	3300	175	0.245	36.5	9208	11363	442	4387	251554
32	2900	225	0.219	30	19950	9988	2692	9936	316898
33	3800	100	0.073	35	16834	6704	1205	11897	270513
34	3900	250	0.84	35	13298	8004	1275	10973	273123
35	4200	275	0.06	28	12317	6188	1041	10722	296247
36	4700	150	0.033	34	12604	4217	800	11879	309652
37	5400	75	0.697	38	24497	8343	399	18275	227351
38	5300	250	0.151	27.5	16951	10784	522	10585	254183
39	4000	125	0.035	29	9193	5681	319	14154	240377
40	6000	200	0.126	34	9966	6403	498	16722	215693
41 [b]	6000	200	0.451	36	10526	5708	247	5460	284781
42 [b]	5200	525	0.155	37	17829	8497	467	5843	250540
43 [b]	6200	200	0.047	27	17534	7250	658	5404	269353
44 [b]	5300	950	0.209	22	11700	6381	661	6703	251621
45 [b]	11300	625	0.141	37	12073	12900	371	4455	257977

[a] Lambs ranked according to adult worm count in the total abomasal contents and FEC (eggs per gram wet faeces), at post-mortem.
[b] Lambs selected for the fold-change analysis
[c] anti-*T. circumcincta* IgA concentrations calculated relative to standard curve
