# Supplementary material for: Relationship between susceptibility of Blackface sheep to Teladorsagia circumcincta infection and an inflammatory mucosal T cell response
Source: Vet Res. 2012 Mar 28;43(1):26. doi: 10.1186/1297-9716-43-26 (PMC3422184; doi:10.1186/1297-9716-43-26)
Supplement: Additional file 2 — PCR primers and PCR conditions A. Primers sets based on Bos taurus sequence for amplification of sheep cytokine transcripts. B PCR primers and PCR conditions. [file 1297-9716-43-26-S2.pdf]

Additional file 2
PCR primers and PCR conditions

A. Primers sets based on *Bos taurus* sequence for amplification of sheep cytokine transcripts

Gene	Access. No.	Primer sequence (5' to 3')	Anneal Temp	Primer (nm)	MgCl (mM)	Product Size (bp)
IL7R	XM_599818.4	F: TTCCTGGACTGCCAGATTC<br>R: GAGAGAGTGGGACTCAGTCATC	50°C	500	2.0	537
IL17A	NM_001008412.1	F: CAGCGAGCACAAGTTCATC<br>R: TTGGGGAGTAG8GGGTCAG	55°C	500	3.0	281
IL21	NM_198832.1	F: ACTATGTGAATGACTTGGATCC<br>R: CTAGGACAGATGCTGATGAATC	52°C	500	2.0	537
IL23A	XM_588269.4	F: CAGCTCTCACAGCAACTCTGC<br>R: GGTCAACATCGTCAGTCAGTCAG	55°C	500	2.0	635
IL25	XM_605190.2	F: GAGGAGTGGCTGAAGTGGAAC<br>R: CGGTAGAAGACGGTCTGGTTG	55°C	500	3.0	538

B Primer sequences and conditions used for RT-qPCR of sheep genes

Gene	Access. No.	Primer sequence (5' to 3')	Anneal Temp	Primer (nm)	MgCl (mM)	Product Size	Reaction efficiency	Range of cT values	R^2 value	Mean slope
IL2	NM_001009806	F: CTTCTACATGCCCAAGGTTAACG<br>R: CCTTGATCTCTCTGGTGTTCAGG	62°C	600	2.0	128	1.02	10.68-24.36	0.99	-3.27
IL4	NM_001009313	F: AAACGCCGAACATCCTCAC<br>R: GCCTAAGCTCAATTCCAGTCC	60°C	600	2.0	126	1.09	9.20-22.39	0.99	-3.11
IL6	X68723	F: TCCAGAACGAGTTTGAGG<br>R: CATCCGAATAGCTCTCAG	62°C	500	4.0	236	0.98	14.07-28.65	0.99	-3.37

IL7R	XM_599818	F: CTCCAGGTCTCCTAATGGCA<br>R: CAAGGAAGTGAGGATGGGCT	63°C	600	2.0	172	0.99	11.61-25.90	0.99	-3.35
IL10	U11421	F: CTGTTGACCCAGTCTCTGCT<br>R: ACCGCCTTGCTCTTGTTT	63°C	500	2.0	224	0.95	13.81-28.55	0.99	-3.44
IL12B	AF004024	F: :TCAGACCAGAGCAGTGAGGT<br>R: GCAGGTGAAGTGTCCAGAAT	63°C	500	2.0	243	0.93	13.75-28.37	0.99	-3.49
IL17A	NM_001008412	F: GAAGGCCCACCGATTATC<br>R: GCATTGATACAGCCTGAGTG	62°C	600	2.0	124				
IL21	NM_198832.1	F: CAGCAAATAATGGAGACAACG<br>R: CTCATAAGAATCACAAGAAGGACA	63°C	600	2.0	131	0.94	16.61-31.89	0.99	-3.48
IL23A	FN822243.1	F: ACCTGTGAGCCAATGAGTTC<br>R: GGTCAACATCGTCAGTCAGTC	62°C	700	3.0	93	0.98	12.14-26.74	0.99	-3.37
IL25	NM_001195219	F: TGGCTGAAGTGGAACAGTG<br>R: GACACAGTGTGGACACAGGC	62°C	450	2.5	198	0.99	10.74-24.73	0.99	-3.34
EBI3	EE824867	F: CACATCATTCATTGCCACGTAC<br>R: GCTGTGATGTTGAGCACATAGG	64°C	1000	2.0	147	1.01	19.54-33.91	0.98	-3.28
FOXP3	NM_001144947	F: CTGACAAGGGTTCCTGCTG<br>R: GAGGGTGGCATAGGTGAAAG	64°C	500	2.0	212	0.93	11.32-26.37	0.99	-3.57
IFNG	NM_001009803	F: CTAAGGGTGGGCCTCTTTTC<br>R: CATCCACCGGAATTTGAATC	62°C	250	2.0	237	0.98	16.63-30.64	0.99	-3.36
TGFB1	X76916	F: GAACTGCTGTGTTCGTCAGC<br>R: GGTTGTGCTGGTTGTACAGG	63°C	500	2.0	170	0.96	10.89-25.28	0.99	-3.42
SDHA	NM_174178	F: ACCTGATGCTTTGTGCTCTGC<br>R: CCTGGATGGGCTTGGAGTAA	62°C	300	2.0	126	0.90	10.25-33.09	0.99	-3.60
YWHAZ	AY970970	F: TGTAGGAGCCCGTAGGTCATC<br>R: TCTCTCTGTATTCTCGAGCCATC	62°C	600	3.0	101	0.95	9.98-31.58	0.99	-3.46
