# Supplementary material for: Relationship between susceptibility of Blackface sheep to Teladorsagia circumcincta infection and an inflammatory mucosal T cell response
Source: Vet Res. 2012 Mar 28;43(1):26. doi: 10.1186/1297-9716-43-26 (PMC3422184; doi:10.1186/1297-9716-43-26)
Supplement: Additional file 3 — Normalized copy numbers of cytokine transcripts in abomasal mucosa. [file 1297-9716-43-26-S3.pdf]

Additional file 3
Normalized copy numbers of cytokine transcripts in the abomasal mucosa

Infection rank[a]	Copy numbers per µg total RNA	
	TGFB1	IL6
Uninfected Control<br>mean adult worm count = 0, FEC = 0.		
0	30542	2005
0	33438	451
0	85328	1081
0	18162	765
0	28409	1600
0	28896	774
0	22921	4532
0	19716	2719
0	27460	233
0	27563	1003
Group 1 – Resistant<br>mean adult worm count = 59, FEC = 1.67.		
1	35863	1389
2	19601	15848
3	8086	967
4	39872	882
5	16570	684
6	18653	674
7	15755	677
8	16055	1474
9	21185	782
10	81533	1911
11	35172	1469

12	25147	680
13	27684	742
14	61492	2362
15	44207	803
Group 2 – Intermediate<br>mean adult worm count = 1508, FEC = 82.		
16	18861	649
17	40397	1095
18	36487	1399
19	39243	1050
20	11513	220
21	17602	372
22	30730	826
23	25778	694
24	24269	658
25	24913	431
26	24832	331
27	18108	301
28	22095	399
29	24601	430
30	22645	530
Group 3 - Susceptible<br>mean adult worm count = 5167, FEC = 288.		
31	32431	539
32	23906	473
33	42873	1384
34	46871	4197
35	67306	2480
36	55097	3138

37	36724	193
38	41591	2905
39	26230	1349
40	42629	7536
41	42244	5309
42	55352	4809
43	37461	7360
44	29454	1027
45	30125	1226

[a] Lambs ranked according to adult worm count in the total abomasal contents and FEC (eggs per gram wet faeces), at post-mortem.
